# The impact of ICT goods exports and environmental technology innovation on mineral rents: Evidence from OECD countries

**DOI:** 10.1371/journal.pone.0308143

**Published:** 2024-09-30

**Authors:** Shanshan Dou, Muhan Dong, Junguo Shi, Bert M. Sadowski, Sufyan Sannah Gbolo

**Affiliations:** 1 School of Business, Wuxi Taihu University, Wuxi, China; 2 School of Economics, Northeastern University, Qinhuangdao, China; 3 School of Finance and Economics, Jiangsu University, Zhenjiang, China; 4 School of Innovation Studies, Eindhoven University of Technology, Eindhoven, The Netherlands; Middle Tennessee State University Jennings A Jones College of Business, UNITED STATES OF AMERICA

## Abstract

This study investigates the effects of Information and Communication Technology (ICT) goods exports and environmental technology innovation (ETI) on mineral rents using a panel dataset of 23 OECD countries from 2000 to 2020. Employing a fixed-effects regression and several robustness checks (FGLS, PCSE, and DKSE), we find that ICT goods exports are positively associated with mineral rents, while ETI exerts a negative impact. Notably, the positive effect of ICT goods exports was more pronounced in countries with higher levels of ICT goods exports. Our findings underscore the complex interplay among technological advancements, environmental sustainability, and economic outcomes in resource-dependent economies, emphasizing the need for tailored policy interventions to navigate these multifaceted dynamics.

## 1. Introduction

Amid rapid technological advancements and escalating environmental concerns, the need for innovative policy frameworks within OECD countries has become increasingly urgent (e.g., [1–3]). This study is driven by the critical challenge of harnessing the economic benefits of ICT goods exports, a key driver of modern economic growth, while simultaneously addressing the environmental implications associated with the exploitation of mineral resources [3–5]. The delicate balance between economic prosperity and environmental sustainability poses a complex policy dilemma, particularly in the context of mineral rents, which, while generating substantial national revenues, entails significant risks of greenhouse gas emissions, habitat destruction, and economic volatility [6–8].

Our inquiry into this complex issue is driven by the urgent policy-level problem of designing and implementing strategies to adequately navigate the intricacies of economic growth, technological advancement, and ecological preservation. By examining OECD countries at the crossroads of financial performance, technological progress, and commitment to environmental goals, this study seeks to generate new insights that can inform policymakers in developing robust policies. These policies must not only counteract the adverse environmental impacts of mineral extraction and utilization but also capitalize on the economic prospects offered by the global digital transformation [9, 10]. Our study focuses on OECD countries because of their diverse economic structures, varying levels of reliance on mineral rents, and strong commitments to environmental sustainability, offering fertile ground for examining the complex interplay between ICT, environmental innovation, and resource-based economies [11]. From this perspective, we aim to contribute actionable policy recommendations that can guide sustainable development strategies, ensuring that the economic benefits of ICT goods exports and technological innovations are harnessed without compromising environmental integrity.

ICT exports and environmental technology innovation are increasingly being recognized as potential drivers of sustainable economic transformation, particularly in OECD countries. The imperative for economies to transition towards more sustainable and diversified models is underscored by research highlighting the limitations of traditional, resource-dependent growth paradigms [12, 13]. ICT exports have been identified as crucial facilitators of this transition, with studies demonstrating their significant contributions to GDP growth, job creation, and environmental improvements through enhanced efficiency and resource conservation [3, 14–16]. Furthermore, the OECD’s leading role in ecological technology innovation, exemplified by its investments in renewable energy, pollution control, and waste management solutions [17], reflects a strategic commitment to decoupling economic growth from environmental degradation.

Despite considerable research on the economic impact of ICT goods exports and environmental technology innovation, a significant gap remains in the understanding of their combined effects on mineral rents in the OECD context. This gap underscores a critical policy-level challenge: the development of integrated strategies that maximize the economic benefits of ICT goods exports while minimizing the environmental drawbacks of mineral extraction. This study introduces an analytical framework to explore the intricate relationships among ICT goods exports, environmental technology innovation, and mineral rents. By providing a comprehensive analysis, this study aims to offer actionable insights for policymakers, potentially enabling strategies that other economies could emulate to navigate similar challenges. This effort seeks to deepen our understanding of the delicate balance between economic resilience and environmental sustainability in an era marked by rapid technological progress.

This study addresses two pivotal research questions: To what extent do exports of ICT goods influence mineral rents in OECD countries? Second, what are the effects of ETI on these rents? These questions are crucial for understanding the role of ICT in the digital economy and the importance of mineral rents as a significant revenue source. Furthermore, the development of environmental technologies is essential to mitigate the adverse environmental impacts of mineral extraction. We used panel data from 23 OECD countries from 2000–2020 to answer both research questions by applying a fixed-effects regression model. In this way, this research provides empirical evidence of the relationship between these factors.

This study makes three significant theoretical contributions to the literature on resource economics and sustainable development. First, it bridges a notable gap in the existing research by providing comprehensive empirical evidence on how ICT goods exports and ETI concurrently influence mineral rents in OECD countries. Unlike previous studies, which often examined these factors in isolation, our integrated approach provides a nuanced understanding of their combined impact on mineral rents, thereby expanding prevailing theories in resource economics. Second, this study unveils a novel analysis of the dual effects of ICT and ETI on the economic pillar of mineral rents, revealing a complex relationship where ICT exports are positively associated with increased mineral rents, whereas ETI exhibits a negative effect. This finding significantly enriches the discourse on balancing technological advancements for economic gains against potential environmental tradeoffs. Lastly, by focusing on the OECD context—a consortium known for its strong economic performance, technological progress, and commitment to ecological sustainability—our study contributes to a deeper understanding of how countries at the forefront of technological innovation and environmental stewardship can navigate the challenges and opportunities presented by ICT exports and ETI. This insight is crucial for policymakers, industry stakeholders, and researchers when formulating strategies to align economic development with environmental sustainability.

The remainder of this paper is organized as follows: Section 2 presents a literature review, Section 3 describes the data and empirical approach, Section 4 discusses the main findings, and Section 5 concludes with policy implications.

## 2. Literature review and hypothesis development

### 2.1 The effect of ICT goods exports on mineral rents

Countries with abundant natural resources tend to grow more slowly than resource-poor countries [18–20]. This "resource curse" of countries with an abundance of natural resources can be traced back to a variety of economic mechanisms (e.g., volatility of commodity prices or failures of economic policy) or political mechanisms (e.g., rent-seeking, weak institutions, or corruption) [7, 20]. However, recent research has indicated that natural resource abundance may hinder ICT development. These effects differ across countries with higher ICT diffusion rates [21].

Recent advancements in ICT have prompted a series of profound structural shifts, encompassing economic restructuring, globalization, heightened capital flows, and an upsurge in the utilization of information [22]. The export of ICT goods is a crucial and rapidly growing component of many economies, with substantial economic development and environmental sustainability implications [3]. These exports, which are pivotal for economic diversification, can reduce resource rents. Countries can shift their economic focus from resource extraction by strategically investing in ICT business development and technological innovation [23], thus minimizing vulnerability to global commodity market fluctuations and fostering sustainable economic growth [24].

A current topic of discussion is the nature of supply chains for ICT goods and their interconnectedness with the global economy, potentially leading to increased international collaboration in sustainably managing mineral resources [25]. Driven by the growing demand for minerals in ICT production, these efforts can contribute positively to resource conservation and environmental sustainability [26]. The surge in ICT goods exports has increased the demand for mineral resources, as ICT devices such as smartphones and computers rely heavily on mineral resources such as rare earth elements and copper [7, 27]. However, this surge in demand has raised concerns regarding the sustainability of mineral resources on a global scale [8].

Exports of ICT goods drive economic growth in OECD nations, significantly contributing to Gross Domestic Product (GDP) and job creation, thus enhancing economic prosperity [28, 29]. They improve the trade balance and foster innovation and competitiveness in various sectors. These exports have the potential to influence mineral rents and foster economic development. Integrating advanced technologies within the mining industry, such as automation, data analytics, and remote monitoring systems, which are applications and services driven by the ICT industry, has led to significant improvements in resource management. [9] indicated that adopting digital technology in the mining industry has improved productivity by optimizing processes and reducing human error, potentially leading to cleaner technologies and more sustainable mineral extraction practices. To better understand how the export of ICT goods directly affects mineral rents, it can be assumed that increased demand for specific minerals is crucial for ICT manufacturing, thereby influencing mineral rents. Based on the above discussion, we propose Hypothesis 1.

**Hypothesis 1: Exports of ICT goods have a positive impact on mineral rents**.

### 2.2 The effect of environmental technology innovation on mineral rents

Progress towards ecological sustainability is vital for further economic and industrial development [30]. This literature has converged on the assumption that new technologies, market-based solutions, and regulations on their own are inadequate for achieving strong sustainability of natural resources [31]. More complex solutions are required to address long-term sustainability challenges with new innovative technologies [32].

Environmental technological innovation is pivotal for mitigating the adverse impacts of manufacturing processes and products on the natural environment. This encompasses a range of techniques, such as pollution prevention, waste recycling, carbon capture technologies, hydrogen generation, energy conservation, nanotechnology, and environmental management [33, 34]. A key aspect of ETI is its potential to promote a shift from extractive industries towards cleaner and more sustainable technologies [11, 35]. For example, the growing adoption of electric vehicles reduces the demand for fossil fuels and impacts the associated mineral resources, such as oil and gas. This shift poses economic challenges for mineral-rich economies that rely heavily on non-renewable resources [36]. Advancements in materials science, leading to the substitution of traditional minerals with sustainable alternatives in green technology production, exemplify resource efficiency driven by ETI [37, 38].

The development and implementation of ETI have lowered the demand for specific mineral resources and reduced mineral rents. The rise of renewable energy sources, such as wind and solar power, for instance, has decreased reliance on fossil fuels, impacting the mineral rents of countries dependent on these conventional energy sources [39]. This transition is also influenced by stricter environmental regulations, which, while necessary for sustainable resource management, increase compliance costs for mining companies and can affect their profitability, and subsequently, mineral rents [40–42]. However, adopting cleaner production techniques in mining, such as advanced waste management systems and energy-efficient processes, can significantly reduce environmental footprints and enhance resource efficiency [43, 44]. These technological innovations minimize the release of harmful pollutants and contribute to sustainable mining practices. Research on green technologies and mineral resource consumption has emphasized the potential of recycling technologies to recover valuable metals from electronic waste, thereby reducing the need for primary mineral extraction [45, 46]. This trend suggests that the widespread adoption of green technologies could lead to more sustainable resource consumption patterns, thereby affecting mineral revenue. As a result of this discussion, Hypothesis 2 is formulated.

**Hypothesis 2: Environmental technological innovation negatively impacts mineral rents**.

## 3. Variables, data, and model

### 3.1 Variables

We measured the explained variable using mineral rents over total natural resource rents (MRTN). Mineral rents are the surplus from mineral production, expressed as a percentage of GDP, while total natural resource rents encompass the summation of oil, natural gas, coal, mineral, and forest rents, expressed as a percentage of GDP. This is in line with recent studies [7, 8].

The explanatory variables are environmental technology innovation (ETI) and information and communications technology goods exports (ICT). The percentage of total ICT goods exports measures the competitiveness of ICT in a country [3]. It includes computers, peripheral equipment, communication equipment, consumer electronics, electronic components, and other information and technology goods. ETI is measured by environmental inventions over all technologies.

The control variables used in the analysis are GDP per capita (GDP), merchandise trade (MT), the consumer price index (CPI), employment (EP), and adjusted savings due to energy depletion (EN). To mitigate the potential estimation bias arising from significant variations across countries, this study applied a logarithmic transformation to the variables EP, GDP, and EN.

### 3.2 Model specification and estimation method

We built a fixed-effects panel data model, which excels in handling data with individual heterogeneity, temporal dynamics, and inter-individual variations. Eq (1) shows the regression equation.

$$MRTN_{it}=a_{0}+a_{1}ICT+a_{2}ETI+a_{3}{GDP}_{\mathrm{it}}+a_{4}{MT}_{\mathrm{it}}+a_{5}{EN}_{\mathrm{it}}+a_{6}{CPI}_{\mathrm{it}}+a_{7}{EP}_{\mathrm{it}}+C_{i}+T_{t}+u_{it}$$ (1)

where *MRTN_it_* is the dependent variable for country *i* at time *t*. *a*_0_ represents a constant term, *ICT* and *ETI* are independent variables. *a*_1_ and *a*_2_ denote coefficients of these independent variables. The notations *MT*, *EN*, *CPI*, and *EP* denote control variables. A fixed-effects regression is particularly suitable for our research context, as it effectively controls for unobservable, time-invariant, country-specific factors (e.g., institutional quality, natural resource endowment) that could be correlated with mineral rents. *C_i_* and *T_t_* are the country and year effect respectively.

Furthermore, we employed the Hausman test to compare the appropriateness of the fixed- and random-effects models. The result of the Hausman test supports our choice of using the fixed-effects model for regression analysis.

To further enhance the accuracy of our estimation results, we conduct relevant tests on the data. Through the Pesaran cross-sectional dependence (CD) test, if the p-value is below the significance threshold (e.g., 0.05), we can reject the null hypothesis, indicating the presence of cross-sectional dependence in the data. Employing Pesaran’s CADF test for second-generation unit root tests suggests that the data may contain a unit root, indicating that the series could be non-stationary. Considering the issues of heteroscedasticity and autocorrelation in macroeconomic data, in Section 4.2, we also use feasible generalized least squares (FGLS), panel-corrected standard error (PCSE), and Driscoll-Kraay standard errors (DKSE) to ensure the robustness of our regression results.

Moreover, recognizing that the relationship between ICT goods exports and mineral rents may not be uniform across all countries, this study employs a heterogeneity analysis. This analysis stems from the understanding that countries with varying levels of ICT sector development may experience different impacts from changes in ICT goods export. To investigate this, we divide the sample into two groups: those with "higher ICT goods exports" and those with "lower ICT goods exports." This grouping is achieved using a k-means clustering algorithm that identifies a natural division or clustering pattern within the data based on the distribution of ICT goods export values. By separating countries into these two groups, the analysis can then assess whether the effect of ICT goods exports on mineral rents differs significantly.

### 3.3 Data processing and description

In this study, we selected OECD countries as the research sample because of their advanced economies, well-established ICT competitiveness, and strong commitments to environmental sustainability. These factors make them ideal candidates for examining the impact of ICT goods exports and ETI on mineral rents. The OECD countries present a spectrum of mineral resource dependencies and diverse environmental policies, providing a rich context for our analysis. Furthermore, the availability of consistent and reliable data on ICT goods exports, ETI, and mineral rents is crucial in the selection process.

For this study, indicators such as ETI, GDP, CPI, EP, and EN are taken from OECD Statistics, and others are taken from the World Bank Development Indicator. The study period was from 2000 to 2020.

During data pre-processing, we excluded observations in which mineral rents were recorded as zero. This decision was made to ensure data integrity and maintain research quality, as zero values could indicate data reporting errors or anomalies that deviate from typical patterns. This exclusion, while potentially limiting the scope of the study to cases of non-existent or negligible mineral rents, was essential for preserving the internal consistency of our research.

Our sample includes 24 OECD countries: Australia, Austria, Canada, Chile, Denmark, France, Germany, Greece, Hungary, Ireland, Israel, Italy, Japan, Korea, Mexico, New Zealand, Norway, Poland, the Slovak Republic, Spain, Turkey, the United Kingdom, and the United States. This selection, encompassing a wide range of economic and environmental policy landscapes, provides a comprehensive cross-sectional view for our analysis.

Table 1 summarizes the variables. The United Kingdom has an MRTN value close to zero, indicating relatively low dependence on mineral resources. In contrast, Chile had the highest MRTN of over 90%, demonstrating heavy reliance on mineral extraction. Colombia and Chile exhibit lower values for ICT goods exports, suggesting a less advanced ICT status. Meanwhile, Ireland, Korea, and Hungary were significantly led by exports of ICT products. Regarding ETI, Ireland, the Slovak Republic, and Austria are recorded higher figures, implying greater focus on and investment in curbing environmental pollution in these countries.

**Table 1:** Descriptive statistics.

Variable	Obs	Mean	Std.Dev.	Min	Max
MRTN	405	0.203	0.26	0.000	0.972
ICT	405	7.405	7.297	0.098	33.703
ETI	405	0.406	0.726	0.005	7.603
GDP	405	10.309	0.44	9.121	11.406
CPI	405	0.999	0.167	0.318	2.344
EP	405	9.388	1.203	7.52	11.967
MT	405	0.569	0.311	0.172	1.756
EN	405	20.316	2.66	13.384	25.656

Table 2 presents the results of the correlation matrix. All the correlation coefficients were lower than 0.7, confirming the absence of multicollinearity among the main explanatory variables.

**Table 2:** Correlations matrix.

	MRTN	ICT	ETI	GDP	MT	EN	CPI	EP
MRTN	1							
ICT	-0.219*	1						
ETI	-0.001	0.036	1					
GDP	-0.130*	-0.099	0.315*	1				
MT	-0.066	0.372*	0.233*	-0.096	1			
EN	-0.264*	-0.156*	-0.275*	0.154*	-0.388*	1		
CPI	0.210*	-0.122	0.101	0.302*	0.073	0.050	1	
EP	-0.135*	0.183*	-0.261*	-0.029	-0.466*	0.397*	0.098	1

Note: After Bonferroni adjustment, a star (*) denotes a significant correlation at the 1% level.

## 4. Regression results and discussion

### 4.1 Basic regression results

Table 3 presents the results of the fixed-effects generalized least-squares (FE-GLS) regression estimation. Model 1 displays the outcomes without including the key independent variables. Model 2 adds all variables to the equation and demonstrates the comprehensive effects of ICT goods exports and ETI on mineral rents. All models controlled for the effects of country and year. The fitness of the models was evaluated using R-squared and Wald chi-square tests.

**Table 3:** Regression results.

	Model 1	Model 2
GDP	0.250**	0.429***
	(0.103)	(0.106)
MT	-0.016	-0.148*
	(0.086)	(0.082)
EN	-0.053***	-0.031***
	(0.012)	(0.012)
CPI	0.220***	0.139**
	(0.069)	(0.067)
EP	-0.384***	-0.728***
	(0.145)	(0.143)
ICT		0.009***
		(0.002)
ETI		-0.097***
		(0.016)
_cons	2.066**	3.126***
	(1.131)	(1.080)
F	5.24***	7.94***
R-squared	0.031	0.017
N	405	405

Standard errors in parentheses; Fixed-effects model with robust standard errors; Year and country fixed effects are included in each model.

* *p* < 0.1

** *p* < 0.05

*** *p* < 0.01

The positive and statistically significant coefficient of ICT provides strong evidence that ICT goods exports positively impact mineral rents. Thus, Hypothesis 1 is supported. First, ICT enhances the efficiency and productivity of mining operations by using advanced technologies to optimize extraction processes and reduce costs [9]. Second, it enables better connectivity and communication among various stakeholders within the mining industry, thereby promoting collaboration and innovation [25]. Third, ICT is vital to environmental and sustainability practices, allowing for better monitoring and management of ecological impacts. This positive environmental performance can enhance mining companies’ social acceptance and reputation and may contribute to higher mineral rents. Additionally, integrating ICT and environmental technologies, such as renewable energy systems and waste treatment technologies, can enable more sustainable mining practices, leading to higher mineral rents.

The variable ETI yields a significant negative coefficient at the 1% level. This indicates that ETI has a negative effect on mineral rents. Thus, Hypothesis 2 is confirmed. This indicates that higher degrees of environmental technological innovation are related to lower mineral rents.

### 4.2 Robustness check

Table 4 shows the results of the robustness analysis. The test begins with Column (1), which applies feasible generalized least squares (FGLS) to the panel data model, accommodating the estimation of serial correlation. In column (2), the panel-corrected standard error (PCSE) estimates are computed for linear cross-sectional time-series models. This method adjusts for potential heteroscedasticity and autocorrelation in the panel data. Column (3) is based on an estimation using the Driscoll-Kraay standard errors (DKSE). Based on the findings, the coefficients of ICT and ETI are statistically significant in each model and consistent with the primary results shown above.

**Table 4:** Robustness test.

	FGLS	PCSE	DKSE
ICT	0.014***	0.015***	0.009***
	(0.003)	(0.004)	(0.002)
ETI	-0.060***	-0.059**	-0.097***
	(0.015)	(0.033)	(0.020)
GDP	0.175*	0.170	0.429***
	(0.121)	(0.190)	(0.109)
MT	0.118	0.121	-0.148*
	(0.115)	(0.125)	(0.101)
EN	-0.041***	-0.040***	-0.031***
	(0.011)	(0.015)	(0.010)
CPI	0.003	0.005	0.139*
	(0.094)	(0.068)	(0.102)
EP	-0.355***	-0.357	-0.728***
	(0.171)	(0.339)	(0.198)
_cons	2.717***	2.760	3.126***
	(1.366)	(2.657)	(1.384)
N	405	405	405
Wald Chi2	515.87***	1612292.24***	-
F	-	-	4057981.23***
R-squared	-	0.5951	0.358

Standard errors in parentheses: Year and country fixed effects are included in each model.

* p < 0.2

** p < 0.1

*** p < 0.05

### 4.3 Heterogeneity analysis

The heterogeneity analysis, presented in Table 5, delves into the nuanced relationship between ICT goods exports and mineral rents by dividing the sample into two groups based on the level of ICT goods exports. This grouping was achieved using a k-means clustering algorithm, which identified a natural division in the data, separating countries with "higher ICT goods exports" from those with "lower ICT goods exports." This approach allows us to examine whether the impact of ICT goods exports on mineral rents varies depending on a country’s relative position in the global ICT landscape. Understanding such potential heterogeneity is crucial for policymakers, as it could suggest that policies designed to foster ICT sector growth may have differential effects on mineral rents, depending on a country’s existing level of ICT development. Although PCSE estimation is a valuable robustness check in panel data analysis, its application is not compatible with the k-means clustering approach used in our heterogeneity analysis; therefore, these results are not presented.

**Table 5:** Regression results among different ICT goods export levels.

	Higher ICT goods export			Lower ICT goods export		
	FE-GLS	FGLS	DKSE	FE-GLS	FGLS	DKSE
ICT	0.020***	0.024***	0.020***	0.004	0.003	0.004
	(0.007)	(0.006)	(0.006)	(0.004)	(0.004)	(0.004)
ETI	-0.055***	-0.027	-0.055**	-0.304***	-0.292***	-0.304**
	(0.020)	(0.017)	(0.026)	(0.099)	(0.076)	(0.133)
GDP	0.313***	0.091	0.313**	0.501	0.543*	0.501
	(0.116)	(0.123)	(0.119)	(0.396)	(0.321)	(0.421)
MT	0.595***	0.580***	0.595***	0.152	0.091	0.152
	(0.168)	(0.172)	(0.166)	(0.205)	(0.157)	(0.241)
EN	-0.023	-0.039***	-0.023**	-0.093***	-0.099***	-0.093***
	(0.014)	(0.013)	(0.010)	(0.022)	(0.017)	(0.014)
CPI	0.128*	0.011	0.128	0.084	0.185	0.084
	(0.069)	(0.098)	(0.112)	(0.263)	(0.220)	(0.212)
EP	-0.477***	-0.221	-0.477**	0.120	-0.065	0.120
	(0.177)	(0.199)	(0.215)	(0.409)	(0.332)	(0.465)
_cons	1.742	2.117	1.381	-3.900	-3.117	-4.354
	(1.213)	(1.519)	(1.273)	(5.499)	(4.125)	(5.877)
R2	0.8676	-	0.4571	0.8215	-	0.4934
F	-	-	4.89e+08***			14402.06***
Wald Chi2	-	509.60***		-	604.19***	-
N	304	304	304	101	101	101

Standard errors in parentheses; Fixed-effects model with robust standard errors.

* p < 0.1

** p < 0.05

*** p < 0.01

The regression results provide compelling evidence of a threshold effect on the impact of ICT goods exports on mineral rents. For countries with higher ICT goods exports, the coefficient of ICT is positive and statistically significant, indicating that increases in ICT goods exports are associated with a corresponding increase in mineral rents. This suggests that in these nations, a more robust ICT sector, likely characterized by greater innovation and efficiency, translates into a greater capacity to leverage ICT for gains in mineral rents earnings. However, for countries with lower ICT goods exports, the coefficient, while still positive, was not statistically significant. This implies that, below a certain threshold of ICT sector development, the positive effects of ICT goods exports on mineral rents may be muted or less apparent.

The heterogeneity analysis presented in Table 5 reveals intriguing insights into the relationship between ICT goods exports and mineral rent. Specifically, the analysis suggests that the positive impact of ICT goods exports on mineral rent is more pronounced in countries with higher ICT goods exports. In such economies, ICT goods exports exhibit a statistically significant positive coefficient, indicating that an increase in ICT goods exports is associated with a corresponding increase in mineral rents. Conversely, in countries with lower ICT goods exports, the positive effect of ICT on mineral rents lacks statistical significance. This observation suggests a potential threshold effect, in which a certain level of ICT sector development may be necessary for ICT goods exports to exert a substantial influence on a nation’s mineral rent earnings. This could be attributed to a more developed ICT sector, enabling greater efficiency and innovation in resource extraction, thereby boosting mineral rent.

The analysis of ETI reveals a consistently negative and statistically significant impact on mineral rents across both groups, suggesting that advancements in environmental technologies are associated with a decline in mineral rent earnings. However, the magnitude of this negative effect is notably larger for the "lower ICT goods export" group. This indicates that countries with less developed ICT sectors may experience a sharper decrease in mineral rents in response to environmental technology innovation. This stems from these economies’ reliance on traditional resource extraction methods, which are often less environmentally friendly. As a result, they are more susceptible to the economic impacts associated with the transition to greener technologies.

### 4.4 Discussion of results

This study provides valuable empirical evidence of the complex relationship among ICT goods exports, ETI, and mineral rents in OECD countries, offering insights for policy decisions. Unlike previous studies that have often examined these factors in isolation, our integrated approach reveals a more nuanced understanding of their combined impact on mineral rents. We find that ICT goods exports have a significant positive effect on mineral rents, particularly in countries with high levels of ICT goods exports. This supports the idea that a thriving ICT sector, characterized by innovation and efficiency, can enhance a nation’s capacity to leverage its mineral resources for economic gain, aligning with findings from studies such as [28, 29] on the positive economic contributions of ICT. Conversely, our analysis revealed a consistently negative relationship between ETI and mineral rents across all OECD countries, suggesting a potential trade-off between environmental innovation and economic gains from mineral extraction.

These findings have important implications for policymakers in OECD countries, navigating the complex challenge of balancing economic growth and environmental sustainability. As highlighted by [19], resource dependency hinders sustainable development. Our research suggests that promoting ICT sector growth, particularly in countries with established ICT infrastructure, can lead to increased mineral rents, potentially enhancing economic prosperity. However, policymakers must also recognize the potential economic consequences of transitioning to greener technologies, as evidenced by the negative impact of ETI on mineral rents. This highlights the need for comprehensive policy frameworks that incentivize the adoption of environmental technologies while mitigating potential adverse economic impacts on the mineral extraction sector. This aligns with the recommendations of [47], who advocates policy innovation to address the challenges of climate change and sustainable development.

This study makes several contributions to the literature. First, it bridges a notable gap in the literature by providing comprehensive empirical evidence on how ICT goods exports and ETI concurrently influence mineral rents in OECD countries. This integrated approach challenges and expands on the prevailing theories in resource economics, which often present a simplistic trade-off between economic growth and environmental sustainability. Second, this study unveils a novel analysis of the dual effects of ICT and ETI on mineral rents, revealing a complex relationship where ICT exports are positively associated with increased mineral rents, whereas ETI exhibits a negative effect. This finding significantly enriches the discourse on balancing technological advancements for economic gains against potential environmental tradeoffs. Lastly, by focusing on the OECD context—a consortium known for its strong economic performance, technological progress, and commitment to ecological sustainability—our study contributes to a deeper understanding of how countries at the forefront of technological innovation and environmental stewardship can navigate the challenges and opportunities presented by ICT exports and ETI. This insight is crucial for policymakers, industry stakeholders, and researchers when formulating strategies to align economic development with environmental sustainability.

## 5. Conclusions and policy implications

This study reveals a complex relationship between ICT goods exports, environmental technology innovation, and mineral rents in OECD countries, moving beyond the simple paradigm of trade-offs between economic gains and environmental stewardship. Our findings highlight a potential threshold effect for ICT goods exports. In countries with a more robust ICT sector, characterized by higher levels of ICT goods exports, we observe a significant and positive association between ICT advancements and mineral rents. Conversely, the impact of ETI on mineral rents is consistently negative across all OECD countries examined, although the magnitude of this effect is amplified in countries with less-developed ICT sectors.

Our findings illuminate the dual role of ICT goods exports and ETI in influencing mineral rents in OECD countries, presenting a nuanced landscape for policy formulation. The positive impact of ICT exports on mineral rents underscores the potential of such exports to serve as a lever for economic growth, suggesting that OECD countries should integrate advanced ICT infrastructure and digital innovation into their economic development strategies. This integration could involve policies aimed at enhancing ICT skills in the workforce, fostering innovation ecosystems that support ICT startups and SMEs, and investing in digital infrastructure that enables efficient resource management and access to global markets. Conversely, the negative association between ETI and mineral rents highlights a critical trade-off between environmental sustainability and economic benefits derived from mineral extraction. This finding calls for a balanced policymaking approach in which the promotion of ETI is aligned with measures to mitigate its potential adverse economic impacts. Policies could include incentivizing research and development in cost-effective environmental technologies, establishing public-private partnerships to share the financial risks associated with adopting new technologies, and creating frameworks that encourage the adoption of green technologies without disproportionately burdening the mineral extraction sectors.

Furthermore, the OECD context, characterized by advanced economies committed to environmental sustainability, provides fertile grounds for implementing policies that harmonize economic growth with ecological preservation. Policy initiatives should prioritize technological innovations that directly align with and advance development goals. This may involve regulatory frameworks that encourage sustainable mining practices, fiscal incentives for companies to adopt environmentally friendly technologies, and international cooperation to set global standards for sustainable mineral extraction and technology use. In conclusion, the policy implications of our study suggest a proactive stance in leveraging ICT for economic development while carefully navigating the complexities introduced by ETI in mineral rent. This approach enables OECD countries to benefit from technological advancements that support both economic prosperity and environmental sustainability.

Our findings contribute to the existing body of knowledge by offering empirical evidence on the complex interplay between ICT goods exports, environmental technology innovation, and mineral rents, challenging and expanding upon prevailing theories in resource economics. Although this study has significantly advanced our understanding of the interplay between mineral rents, ICT goods exports, and ETI, numerous opportunities for further research remain. Future research should delve into the types of ICT goods and environmental technologies that most significantly impact mineral rents, and explore the role of varying global contexts and different minerals in these relationships. This may encompass examining institutional factors such as the role of government policies and regulations, or economic elements such as market conditions and industry competition.

Moreover, future research could benefit from longitudinal studies to unravel the temporal dynamics governing these relationships, offering a clearer picture of the enduring effects of ICT goods exports and ETI on mineral rent. Finally, a valuable extension of this study could involve scrutinizing these relationships across diverse geographical contexts or among various mineral types. This would help ascertain whether the conclusions drawn from this study are universally applicable or are more narrowly confined to OECD countries and the specific minerals analyzed in this study. In our study’s examination of the complex interplay between ICT exports, environmental technology innovation, and mineral rents across OECD countries, we recognized the potential value of conducting sub-sample analyses. Such differentiated analyses promise to unveil more nuanced insights into the diverse effects these variables may have in distinct contexts, reflecting the heterogeneous nature of OECD countries. Future research endeavors with access to larger datasets are encouraged to explore these avenues as they have the potential to contribute substantially to our understanding of these dynamics. Such investigations would undoubtedly enhance the granularity of policy recommendations, tailoring them to the specific economic and environmental contexts of different subsets of OECD countries.
